# Comparative Analysis of Seed Transcriptome and Coexpression Analysis Reveal Candidate Genes for Enhancing Seed Size/Weight in *Brassica juncea*

**DOI:** 10.3389/fgene.2022.814486

**Published:** 2022-02-24

**Authors:** Shikha Mathur, Kumar Paritosh, Rajesh Tandon, Deepak Pental, Akshay K. Pradhan

**Affiliations:** ^1^ Department of Genetics, University of Delhi South Campus, New Delhi, India; ^2^ Centre of Genetic Manipulation of Crop Plants, University of Delhi South Campus, New Delhi, India; ^3^ Department of Botany, University of Delhi, New Delhi, India

**Keywords:** *Brassica juncea*, seed size, thousand seed weight, transcriptomics, RNA-seq, coexpression

## Abstract

Seed size/weight is a multigenic trait that is governed by complex transcriptional regulatory pathways. An understanding of the genetic basis of seed size is of great interest in the improvement of seed yield and quality in oilseed crops. A global transcriptome analysis was performed at the initial stages of seed development in two lines of *Brassica juncea*, small-seeded EH-2 and large-seeded PJ. The anatomical analyses revealed significant differences in cell number and cell size in the outer layer of the seed coat between EH-2 and PJ. Pairwise comparisons at each developmental stage identified 5,974 differentially expressed genes (DEGs) between the two lines, of which 954 genes belong to different families of transcription factors. Two modules were found to be significantly correlated with an increased seed size using weighted gene coexpression network analysis. The DEG and coexpression datasets were integrated with the thousand seed weight (Tsw) quantitative trait loci (QTL) mapped earlier in the EPJ (EH-2 × PJ) doubled haploid (DH) population, which identified forty potential key components controlling seed size. The candidate genes included genes regulating the cell cycle, cell wall biogenesis/modification, solute/sugar transport, and hormone signaling. The results provide a valuable resource to widen the current understanding of regulatory mechanisms underlying seed size in *B. juncea*.

## Introduction

Seed size/weight is one of the most important traits from the evolutionary and breeding perspectives, as it is directly related to the overall yield of a crop. Identification of factors for regulation of seed size is a major challenge for crop improvement programs, particularly in oilseed crops where the seed is the major source of the product. Seed size is determined by coordinated cell proliferation and/or cell expansion in the triploid endosperm, the diploid maternal tissue of the ovule, and the diploid embryo and is controlled by both maternal and zygotic genetic factors (Chaudhury and Berger, 2001; Sun et al., 2010). Seed development in *Arabidopsis thaliana* includes stages of embryogenesis, seed maturation, accumulation of storage compounds, and consumption of free nuclear endosperm by expanding cotyledons followed by rapid desiccation (Sun et al., 2010).

The controlling mechanisms of seed size in plants have been extensively studied in *A. thaliana* and *Oryza sativa* (rice) and include maternally imprinted genes, IKU pathway, ubiquitin–proteasome pathway, mitogen-activated protein kinase (MAPK signaling), G-protein signaling, phytohormones, and transcriptional regulatory factors, which have been well described in some reviews (Li and Li, 2016; Savadi, 2018; Li et al., 2019). It has been shown that the time of cellularization of syncytial endosperm, mitotic activity at initial stages of seed development, and partitioning of carbohydrates and photoassimilates play a major role in the determination of seed size (Borrás et al., 2004; Zhou et al., 2009; Wang et al., 2010; Ruan et al., 2012). It has been suggested that transcriptional trajectories at the early stages of seed development are mostly responsible for the determination of final seed size via restricting cell numbers (Ruan et al., 2012; Du et al., 2017).

A comparative transcriptome analysis of varieties contrasting in seed size at different stages of seed development in *Brassica napus* (Geng et al., 2018), *Brassica rapa* (Niu et al., 2020), soybean (Lu et al., 2016; Du et al., 2017), and maize (Sekhon et al., 2014) has identified promising candidate genes for improvement of the trait. However, no such analysis has been performed to dissect the molecular mechanisms controlling seed size/weight in *Brassica juncea* despite the fact that *B. juncea* germplasm harbors greater variation for seed size than *B. napus* and *B. rapa*. The genomic regions and candidate genes regulating seed size in *B. juncea* have been identified using marker-based bi-parental quantitative trait loci (QTL) mapping or genome-wide association studies (Ramchiary et al., 2007; Dhaka et al., 2017; Sra et al., 2019; Kumar and Bisht, 2020; Kang et al., 2021).

*B. juncea* (AABB) is an important oilseed crop in the Indian subcontinent, which is grown in more than six million hectares during the winter seasons in the northwestern part of the region. Heterosis breeding in *B. juncea* exploits the high genetic diversity between the east European and Indian gene pools to develop hybrid varieties with enhanced yields (Pradhan et al., 1993). Seed size is an important yield influencing parameter in *B. juncea*, and large seed size is desirable to fetch a high market price. The first commercial hybrid of *B. juncea* released in India, DMH -1 (Pusa bold × EH-2), is reported to give a 30% higher yield than the check varieties (Sodhi et al., 2006), but it has a small seed size (thousand seed weight (Tsw) ∼ 3.9 g) as compared to Pusa Bold (Tsw ∼ 6.4 g). The smaller seed size of the hybrid is attributed to the characteristic small seed size of the east European parent-EH-2, which is however a good combiner resulting in high heterosis when crossed with Indian varieties. Therefore, one of the major objectives of genetic improvement of *B. juncea* in India lies in the improvement of the east European gene pool through a precise transfer of some beneficial alleles of seed size from the Indian gene pool without compromising heterosis.

The east European gene pool line, EH-2, and the Indian gene pool line, Pusa Jaikisan (PJ), represent the highest phenotypic variability in seed size in *B. juncea* germplasm reported so far. Dhaka et al. (2017) carried out a QTL analysis of Tsw in *B. juncea* using a bi-parental doubled haploid (DH) population EPJ, derived from F1 of a cross between PJ and EH-2. The study identified twenty-five QTL with the logarithm of odds (LOD) scores greater than 2.5 in the EPJ DH population from three environments. The beneficial alleles in all Tsw QTL were contributed by PJ.

The present study attempts to dissect the transcriptional regulatory pathways controlling seed size/weight in *B. juncea* lines EH-2 and PJ by conducting a comparative transcriptome analysis of the initial developmental stages of seed. The inference of coexpression networks identified coregulated gene sets and hub genes associated with an increase in seed size, which could help deduce the gene regulatory networks underlying the regulation of seed size. Integration of our transcriptome dataset with the known genomic regions associated with Tsw in the EPJ DH population identified putative transcriptional regulators of seed size in *B. juncea*.

## Materials and Methods

### Plant Material and Sample Collection

Two *B. juncea* lines, PJ and EH-2, with contrasting seed weights were grown in the field during the winter season of 2017–2018. Unopened flower buds on the main shoot were artificially self-pollinated and tagged for both the cultivars. Two pods per plant from 10 plants were harvested from each line at 5, 10, 15, 20, 25, and 30 days after pollination (DAP), referred to as stages S0, S1, S2, S3, S4, and S5, respectively. Self-pollinated seeds from 10 biological replicates were harvested at the mature dry stage, referred to as stage S6 from each line for measurement of seed size. At least 120 seeds from each stage were photographed, and seed size was determined using ImageJ (Schneider et al., 2012) software. Tsw and seed oil content of mature dry open-pollinated seeds were measured following Ramchiary et al. (2007). Levene’s test and two-sample *t*-test were used to compare variances and means, respectively.

### Anatomical Analysis

For the anatomical analysis, harvested seeds were fixed in Karnovsky’s fixative (Karnovsky, 1965) for 24 h at 4°C. Fixed seeds were washed and stored in 0.2 M of sodium cacodylate buffer, pH 7.4 at 4°C. The seeds were dehydrated in series with 2-methoxy ethanol, ethanol, and *n*-propanol for 24 h each followed by incubation in *n*-butanol for 12 h. The seeds were subsequently treated with freshly prepared glycol methacrylate monomer twice for 24 h each. The seeds were then embedded in the desired orientation in gelatin capsules containing the fresh monomer, which was allowed to polymerize at 40°C for 24 h followed by subsequent incubations at 50°C, 55°C, and 60°C for 24 h each (Feder and O’Brien, 1968). Sections (2 and 3 µm) of the resin-embedded seeds were cut using a rotary microtome (American Optical Co., United States, model 820) using glass knives. Sections were stained using Toluidine blue O, prepared in sodium benzoate buffer, pH 4.4 (O’Brien and McCully, 1981), mounted in DPX, and then observed under a bright-field microscope. At least 10 seeds sampled at stages S0–S5 from 10 different individuals were analyzed for each cultivar. For seeds sampled at the mature green stage (S5), the cell number and cell size of the outermost epidermal layer of the seed coat were measured. Seeds were dissected to isolate seed coat, which was fixed overnight in FAA at 4°C. The tissue was then cleared by incubation in choral hydrate solution for 2 h. Differential interference contrast (DIC) microscopy was employed to take observations. The desired region of sections was photographed, a number of cells in the outer layer of the seed coat were counted, and the mean area of 500 cells was determined based on ten individuals using the ImageJ software.

### Sample Collection and RNA Isolation

Unopened floral buds on the main shoot were tagged 12–14 h before anthesis and were artificially self-pollinated. Siliques were opened using fine forceps/needles, and intact seeds were immediately frozen in liquid nitrogen. Samples were harvested from both PJ and EH-2 on the same day at approximately mid time of photoperiod, within a 2-h interval (1,100–1,300 h) for each stage. As the seed size is known to vary with the branch position in *B. napus* (Ren et al., 2017), seeds were collected from different positions (whorls) on the main shoot of five individuals for each stage. An equal number of seeds were pooled from each of the five individuals for one biological replicate. Seeds were collected in three biological replicates at stages S1–S3 and were stored at −80°C until further processing.

Total RNA was isolated from EH-2 and PJ seeds, using Spectrum Plant Total RNA kit (Sigma-Aldrich, St. Louis, MO, United States) following the manufacturer’s protocol. Each sample was given on-column DNase 1 treatment using RNase-free DNase (Qiagen, Valencia, CA, United States). RNA samples were checked for genomic DNA contamination by PCR amplification of a housekeeping gene (UBQ9) and were quantified using a NanoDrop spectrophotometer. The integrity of RNA was checked using Bioanalyser (Agilent Technologies, Santa Clara, CA, United States), and RNA samples with RIN ≥ 6 were used for sequencing experiments.

### Transcriptome Sequencing and Quality Assessment

The 18 mRNA libraries (two lines, three stages (S1–S3), and three biological replicates each) were developed from high-quality RNA samples using NEBNext^®^ UltraTM II RNA Library Prep Kit (New England Biolabs, Ipswich, MA, United States) following the manufacturer’s protocol and were sequenced on Illumina platform (HiSeq 10×) (Illumina, San Diego, CA, United States) to generate 150-nucleotide-long paired-end (150 bp ×2) sequence reads. At least 4 GB of data per sample was generated, and the quality of the raw sequence data was assessed using FastQC.^1^ The reads were then filtered for quality, and adapters were removed using Trimmomatic (Bolger et al., 2014) with parameters TruSeq3-PE-2.fa:2:30:10 LEADING:3 SLIDINGWINDOW:4:15 TRAILING:3 MINLEN:60.

### Differential Expression Analysis

The high-quality reads were mapped on the *B. juncea* var. *Varuna* genome sequence (Paritosh et al., 2021) using STAR aligner (Dobin et al., 2013) with default settings. The raw feature counts obtained thereof were normalized using the *variance stabilizing transformation* (vst) as implemented in DESeq2 v1.30.1 (Love et al., 2014) using Bioconductor (Gentleman et al., 2004) in R (R Core Team, 2019) for biological quality assessment and data visualizations. The differential expression analysis was performed on raw feature counts using a generalized linear model with Wald’s statistical test as implemented in the DESeq2 package using the model ∼ *line* + *stage*, where *line* represents the two *B. juncea* lines and *stage* represents the seed developmental stages. The genes with “lfc threshold” of 1 and an adjusted *p*-value ≤0.05 after correcting *p*-value using Benjamini and Hochberg (BH) correction were considered to be significantly differentially expressed.

### Identification of Transcription Factors, Gene Ontology, and Pathway Enrichment

Transcription factors (TFs) were identified using a homology search (blastp, evalue 1e−25) against Plant TF database^2^ (Plant TFDB v5.0). The genes were analyzed for Gene Ontology (GO) enrichment using Blast2GO^3^, and significantly overrepresented GO terms were identified with a false discovery rate (FDR) threshold of <0.1. The enrichment results were summarized using REVIGO^4^ (Supek et al., 2011). KAAS annotation server (Moriya et al., 2007) was used to assign KO terms to *B. juncea* var. Varuna genes using blastp; and hypergeometric test in Blast2GO program was used for pathway enrichment with an FDR threshold of <0.1. Enrichment results were visualized using R package ggplot2 (Wickham, 2011). Additionally, Mercator4 V2.0 (Schwacke et al., 2019) was used to annotate *B. juncea* var. Varuna genes using default parameters, and MapMan was used for pathway enrichment (significance value ≤0.05) of the differentially expressed genes (DEGs).

### Coexpression Network Analysis

Coexpression networks were generated using the weighted gene coexpression network analysis (WGCNA) (Langfelder and Horvath, 2008) package in R to detect modules and key regulatory genes associated with seed size. The vst transformed expression data for expressed genes were used for coexpression analysis, and the genes with very low expression values were excluded from the analysis to avoid spurious nodes. A soft thresholding power of 18 was determined using pickSoftThreshold function based on the scale-free topology model fit (r^2^ > 0.9).

The blockwiseModules function was used to obtain coexpression modules with the following parameters: power, 18; network-type, signed; TOM-type, signed; minModuleSize, 50; maxBlockSize, 60,000; corType, bicor; maxPOutliers, 0.1; and mergeCutHeight, 0.25. mergeCloseModules function at cutHeight = 0.2 was used to merge closely related modules after calculating the dissimilarity of module eigengenes. Pearson’s correlation was used to correlate module eigengenes with trait values. Blast2GO was used for GO enrichment of modules. Cytoscape v3.8.2 (Smoot et al., 2011) was used to visualize the coexpression networks.

### Quantitative PCR Analysis

First-strand cDNA was synthesized from 2 μg of total RNA using High-Capacity cDNA Reverse Transcription Kit (Applied Biosystems, Foster City, CA, United States) according to the manufacturer’s instructions. Real-time quantitative reverse transcription-PCR (qRT-PCR) analyses were carried out using a PowerUp SYBR Green Master Mix (Applied Biosystems, United States) on QuantStudio 6 Flex 210 Real-Time PCR System (Applied Biosystems, United States). Expression profiling of the genes was carried out at stages S1–S3 for EH-2 and PJ to validate the transcriptome data. TIPS-41 gene (Chandna et al., 2012) was used as an internal control. The relative gene expression levels were evaluated following the 2^−ΔΔCT^ method (Livak and Schmittgen, 2001).

## Results

### Phenotypic and Anatomic Variations Between the Two *B. juncea* Lines

**FIGURE 1:**
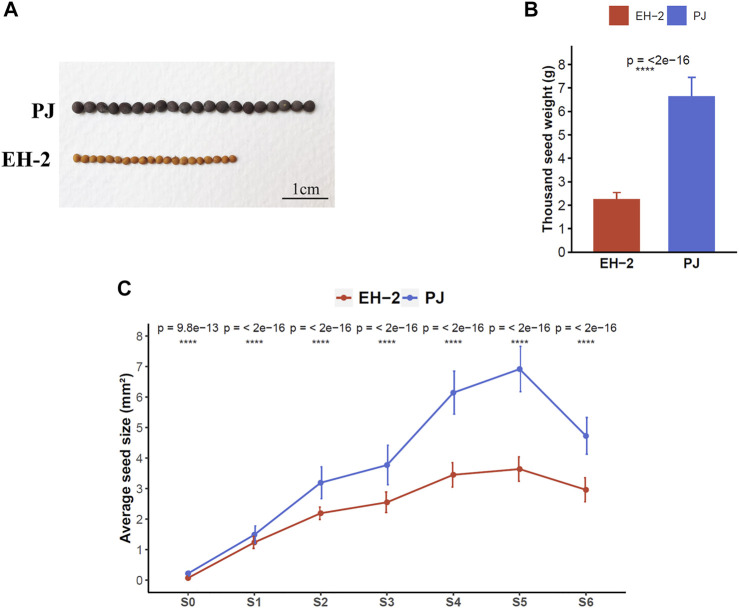
Phenotypic comparisons between the *Brassica juncea* lines EH-2 and PJ. **(A)** Comparisons in seed size and coat color in mature seeds of EH-2 and PJ. **(B)** Comparison of Tsw in mature dry seeds (S6) of EH-2 and PJ. **(C)** Differences in the average seed size of EH-2 and PJ seed sampled at stages S0–S6. Tsw, thousand seed weight.

The two *B. juncea* lines, EH-2 and PJ, showed significant phenotypic contrasts in seed traits including seed size, Tsw, oil content, fatty acid composition, coat color, and glucosinolate content at maturity (Figure 1, Supplementary Table S1). The average seed size and Tsw of mature seed (S6) in PJ (seed size, 4.73 ± 0.6 mm^2^; Tsw, 6.62 ± 0.84 g) was significantly higher than those in EH-2 (seed size, 2.95 ± 0.4 mm^2^; Tsw, 2.24 ± 0.3 g) (Figures 1A,B). Phenotypic measurements for seed size and anatomical analysis were carried out at six stages (designated as S0–S5) encompassing major events in seed development, to correlate seed size with seed developmental stages in EH-2 and PJ (Figure 1C, Supplementary Figure S1). Anatomical analysis revealed stages S1–S3 as the initial stages of embryogenesis with 2- to 4-celled, globular, and transition/heart stage embryos observed at stages S1, S2, and S3, respectively. We observed cellularized micropylar endosperm at the S3 stage (Supplementary Figure S1). Cellularization of syncytial endosperm begins between stages S2 and S3 in both EH-2 and PJ. The fresh seeds of PJ were significantly (*p*-value < 0.05) larger in size (mm^2^) than those of EH-2 at all the six developmental stages identified (Figure 1C). The seed size increased from S0 (0.22 ± 0.07 mm^2^ in PJ and 0.07 ± 0.02 mm^2^ in EH-2) to S5 (6.92 ± 0.74 mm^2^ in PJ and 3.64 ± 0.4 mm^2^ in EH-2) and decreased thereafter due to seed desiccation to a final seed size of 4.73 ± 0.6 mm^2^ in PJ and 2.95 ± 0.4 mm^2^ in EH-2 at S6. The maximum increase in seed size in EH-2 was observed at stages S0–S1 (1.17 ± 0.03 mm^2^) followed by stages S1–S2 (0.96 ± 0.03 mm^2^), and that in PJ was at stages S3–S4 (2.37 ± 0.09 mm^2^) followed by stages S1–S2 (1.7 ± 0.05 mm^2^) (Supplementary Table S1), indicating that the final seed size in *B. juncea* is mostly determined at the early stages of seed development. The average cell size and cell number of the outermost epidermal layer of seed coat in PJ seeds sampled at S5 were greater than in EH-2 (Supplementary Figure S2), showing that differential cell division and cell expansion in seed coat might partly contribute to the observed phenotypic differences in the seed size.

### Analysis of Transcriptome of Early Seed Development in the Two *B. juncea* Lines

As we observed a substantial and differential increase in seed size in EH-2 and PJ through stages S1–S3, a comparative transcriptome profiling of the seeds was performed in triplicates at stages S1, S2, and S3 to identify the factors controlling seed size. More than 252.63 × 10^6^ high-quality reads were generated from a transcriptome sequencing of EH-2 and PJ and mapped to the *B. juncea* var. Varuna genome with an average unique mapping rate of 88.74 (Supplementary Table S2). The raw feature counts (uniquely mapped reads) for each sample were normalized using variance stabilizing transformation (vst) as implemented in the DESeq2 package (Love et al., 2014) for biological quality assessment. Sample PS3c (PJ, S3) clustered with EH-2 stage S3 samples and was excluded from downstream analysis. Pearson’s correlation between the biological replicates at different stages varied from 0.97 to 0.99, indicating high reproducibility among the biological replicates (Supplementary Figure S3A). Principal component analysis (PCA) assigned 17 samples to 6 distinct groups and identified *B. juncea* line from which tissue was sampled and the stage of seed development as the first two principal components explaining 44.1 and 29.23% variance (Supplementary Figure S3B). A total of ∼77.5% of genes were expressed in at least one of the samples.

To validate the expression pattern of DEGs in the transcriptome analysis, a reverse transcription–quantitative PCR (RT-qPCR) analysis was performed for 9 DEGs in EH-2 and PJ at the S1–S3 stages of seed development. The relative expression levels (log2fold change) of the tested genes were found to follow the same expression trend as observed in the RNA-seq data (Supplementary Figure S4), indicating that the transcriptome data accurately reflected the transcript abundance levels.

### Differentially Expressed Genes During Initial Stages of Seed Development

**FIGURE 2:**
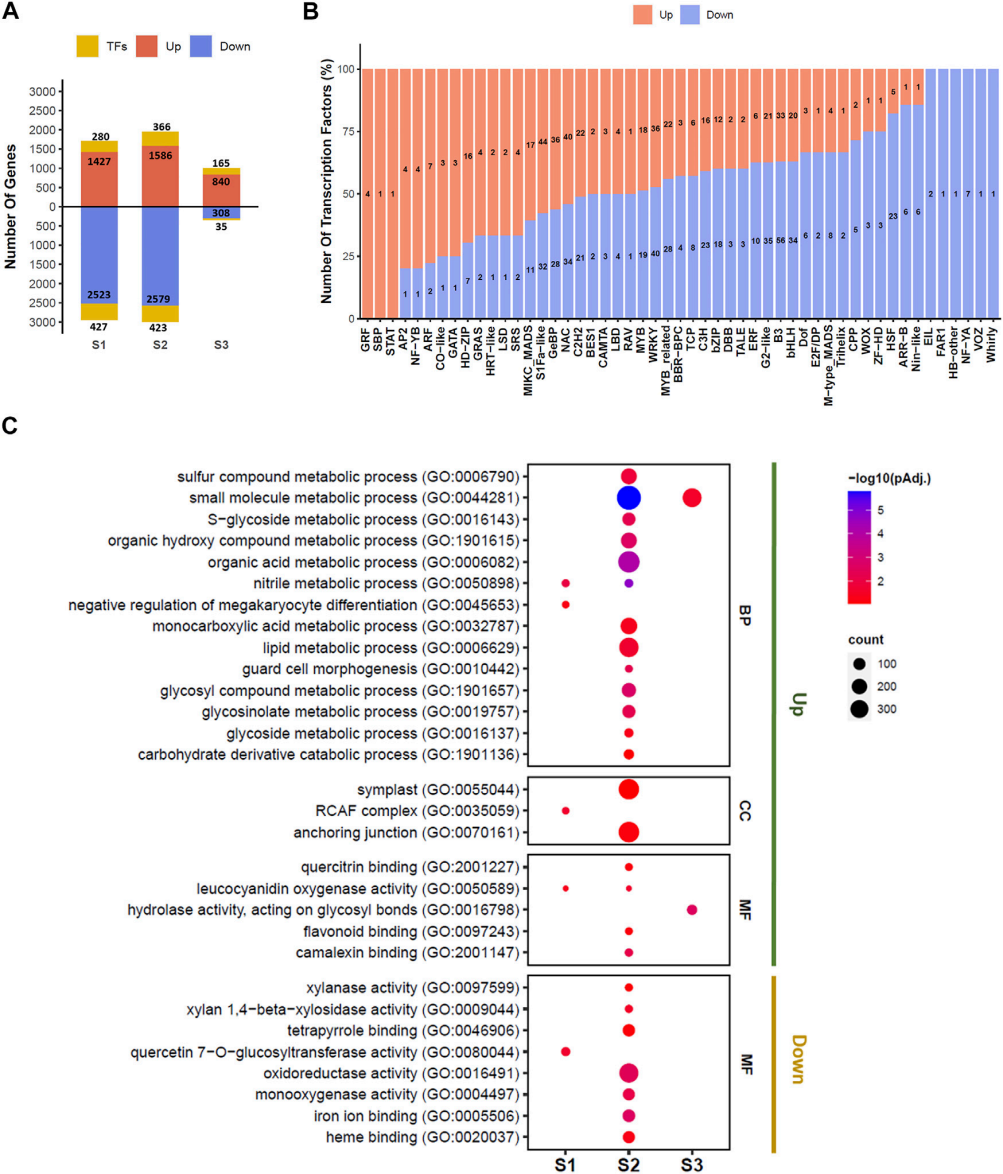
DEGs at different seed development stages between EH-2 and PJ. **(A)** Barplot depicting the number of upregulated and downregulated genes in EH-2 vs. PJ at each developmental stage, and the number of differentially expressed TFs at each stage. **(B)** Barplot depicting the number of TFs belonging to different TF families showing upregulation or downregulation in EH-2 vs. PJ. **(C)** GO enrichment of differentially expressed genes at different seed development stages between EH-2 and PJ. BP, biological process; CC, cellular component; MF, molecular function; E, EH-2; P, PJ; S1–S3, seed development stages; DEGs, differentially expressed genes; TFs, transcription factors; GO, Gene Ontology.

A total of 5,974 genes were significantly differentially expressed (lfcthreshold = 1, padj < 0.05) in pairwise comparisons at the S1, S2, and S3 stages of seed development between EH-2 and PJ (Supplementary Table S3). A total of 2,447 genes including 441 TFs were significantly upregulated, whereas 3,527 genes including 513 TFs were significantly downregulated in EH-2 as compared with PJ at the S1–S3 stages of seed development. The highest number of genes (4,954) was found to be differentially expressed at the S2 stage, and the least number of genes (1,348) was differentially expressed at the S3 stage between EH-2 and PJ (Figure 2A).

A total of 18,596 TFs were identified in *B. juncea* similar to TFs documented in Plant TFDB v5.0 (Supplementary Table S4). Out of these, 954 TFs belonging to 51 TF families were differentially expressed between EH-2 and PJ at the S1–S3 stages of seed development (Figure 2B; Supplementary Table S5). B3, S1Fa-like, WRKY, and NAC TF families seem to play relatively important roles in the initial stages of seed development in *B. juncea*, as 89, 76, 76, and 74 members belonging to these families were differentially expressed in EH-2 and PJ. Members of several TF families, WRKY, ARR-B, GRF, MYB, ARF, B3, AP2, bHLH, bZIP, E2F/DP, MADS, MIKC-MADS, and NAC, were found to be differentially expressed in EH-2 and PJ. These TF families have been implicated in the regulation of seed size in different plant species (Mathew et al., 2016; Li et al., 2019). A total of 7 and 14 TFs belonging to CPP and TCP TF families, respectively, were differentially expressed in EH-2 and PJ (Figure 2B). CPP and TCP TF families regulate organ growth via the regulation of cell proliferation (Yang et al., 2008; Martín-Trillo and Cubas, 2010). Members of the ARF TF family, which regulate auxin-responsive gene expression (Guilfoyle and Hagen, 2007), were upregulated in EH-2, whereas members of the ARR-B TF family, the regulators of cytokinin signal transduction (Sakai et al., 2001), were upregulated in PJ. A total of 64 genes belonging to the GePB TF family of cytokinin-responsive genes (Chevalier et al., 2008) were differentially expressed in EH-2 and PJ. These results suggest that the levels of auxin and cytokinins are differentially regulated during the S1–S3 stages of seed development in EH-2 and PJ. The expression profiles of differentially expressed TFs are given in Supplementary Table S5.

The GO enrichment analysis of DEG revealed several GO terms either commonly or uniquely overrepresented at the S1–S3 stages of seed development (Figure 2C; Supplementary Table S6). The majority of the GO terms were uniquely overrepresented at the S2 stage, suggesting the onset of a major developmental reprogramming of seed development. We observed a consistent overrepresentation of GO terms “lipid metabolic process (GO:0006629)” and “glycosinolate metabolic process (GO:0019757)” in genes upregulated in EH-2 at the S2 stage, suggesting the importance of this stage in differential regulation of fatty acid and glucosinolate content in EH-2 and PJ. The GO terms overrepresented in genes downregulated in EH-2 were “xylanase activity (GO:0097599)” and “xylan 1–4 beta xylosidase activity (GO:0009962),” indicating increased activity of cell wall-modifying enzymes in PJ. The Kyoto Encyclopedia of Genes and Genomes (KEGG) orthology (KO) enrichment analysis revealed pathways differentially regulated in EH-2 and PJ at the S1–S3 stages of seed development (Supplementary Table S7). The KO pathway “Flavonoid biosynthesis (ko00941)” was overrepresented in genes significantly upregulated in PJ, which is consistent with the brown coat color in PJ.

The DEGs between EH-2 and PJ at stages S1–S3 were analyzed using the MapMan tool to further investigate the metabolic pathways and cellular functions differentially regulated in EH-2 and PJ (Supplementary Figure S5). The genes involved in cell wall biosynthesis and modification showed higher expression in PJ at the S1–S2 stages and might be involved in regulating the differences in cell size of seed epidermis as observed in EH-2 and PJ.

### Differential Regulation of Genes Determining Seed Size

**FIGURE 3:**
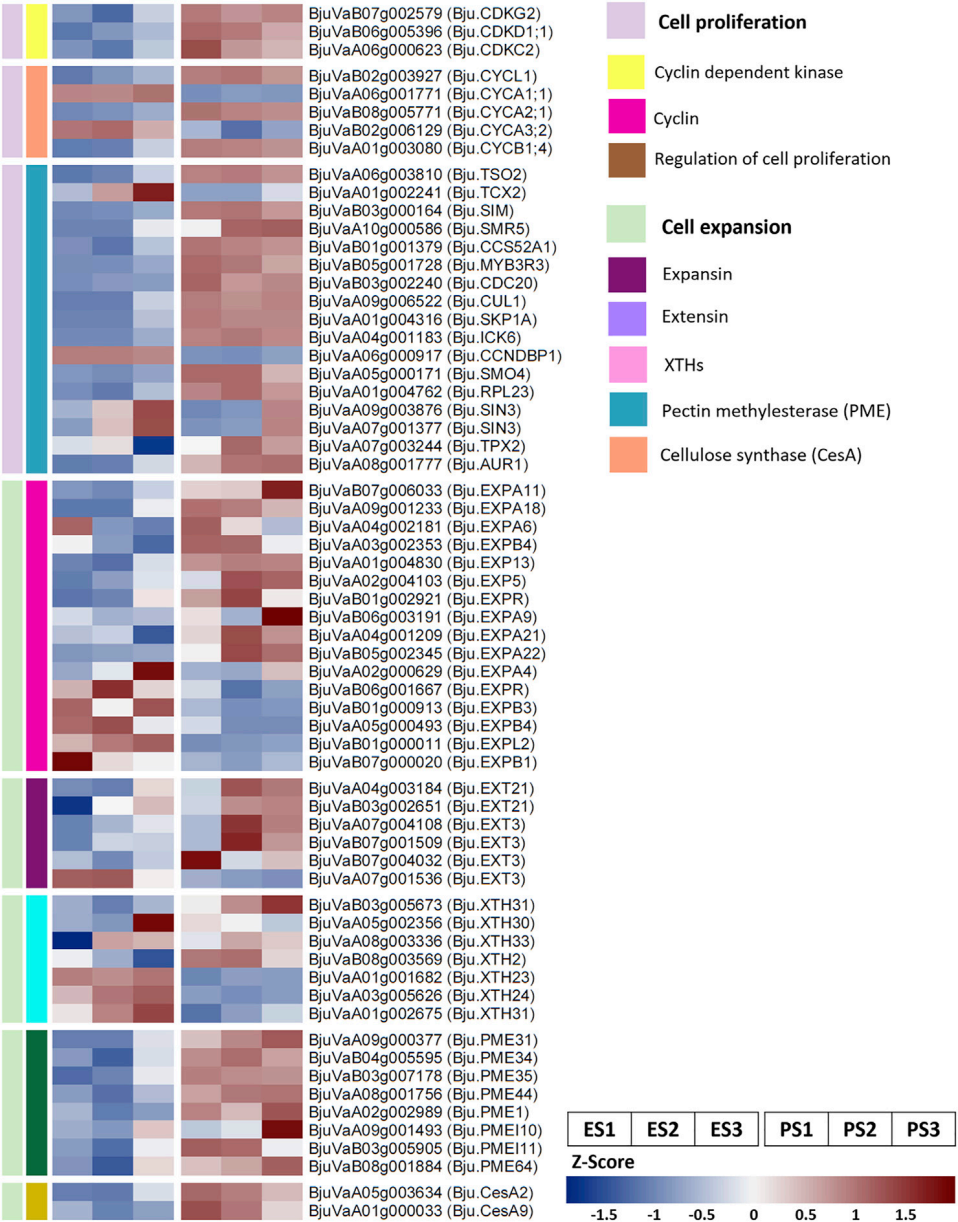
Selected differentially expressed genes in EH-2 and PJ for regulation of cell proliferation and cell expansion. E, EH-2; P, PJ; S1–S3, seed development stages.

We identified the DEGs involved in key cellular processes regulating seed size including cell proliferation, cell expansion, endosperm development, and embryonic development (Figure 3; Supplementary Table S8). In addition, we identified the *B. juncea* homologs of genes reported to regulate seed size in heterologous systems showing differential expression in EH-2 and PJ (Supplementary Table S9).

EH-2 and PJ exhibited differential regulation of cell proliferation at the S1–S3 stages of seed development. Bju.SMO4 (BjuVaA05g000171) was significantly upregulated in PJ at S2. SMO4 is a key regulator of organ growth, and mutation in SMO4 decreases organ size by restricting cell proliferation (Zhang et al., 2015). Interestingly, we found an upregulation of all five paralogs of Bju.AN3 (BjuVaA02g004301, BjuVaA06g003464, BjuVaA09g000483, BjuVaB02g000498, and BjuVaB06g001001) in small-seeded EH-2. AN3 has been reported to influence seed size in *A. thaliana* as a result of increased cell proliferation or cell size (Meng et al., 2016; Liu et al., 2020). Several genes involved in the regulation of the cell cycle were also found to be differentially expressed in EH-2 and PJ. Cyclins Bju.CYCB1;4 (BjuVaA01g003080), Bju.CYCL1 (BjuVaB02g003927), and Bju.CYCA2;1 (BjuVaB08g005771) were upregulated in PJ at S1 and S2. On the other hand, A-type cyclins Bju.CYCA1;1 (BjuVaA06g001771) and Bju.CYCA3;2 (BjuVaB02g006129) were upregulated in EH-2 at S1 and S2. Bju.TSO2 (BjuVaA06g003810) was found to be upregulated in PJ at S1 and S2. It is predominantly expressed in the S phase of the cell cycle and plays a role in dNTP biosynthesis in actively dividing cells (Wang and Liu, 2006). Bju.TCX2 (BjuVaA01g002241) was found to be upregulated in EH-2 at S2. It has been reported as a negative regulator of the cell cycle (Clark et al., 2019). We observed a differential expression of DNA replication machinery in EH-2 and PJ. There was an upregulation of Bju.SIM (BjuVaB03g000164), Bju.SMR5 (BjuVaA10g000586), and Bju.CCS52A1 (BjuVaB01g001379) in PJ at the S1 and S2 stages. SIM and CCS52A1 act together to increase endoreplication in *A. thaliana* trichomes (Kasili et al., 2010).

Gene enrichment analysis revealed increased activity of cell wall modification enzymes in PJ. We found a consistent upregulation of the genes involved in plant cell wall biogenesis that included cellulose synthases (CeSA), xyloglucan endotransglucosylases/hydrolases (XTHs), xyloglucan xylosyltransferase (XXTs), and pectin methylesterases (PMEs) in PJ (Figure 3; Supplementary Table S8). XTHs are implicated in the regulation of cell wall loosening and cell expansion (Rose et al., 2002). We observed a significant upregulation of several genes encoding extensin and expansin proteins in PJ (Figure 3), which also suggests a higher rate of cell expansion in PJ. These data suggest that upregulation of genes is associated with an increase in cell size and cell proliferation in PJ as compared to EH-2 and might explain the higher cell size and cell number in the outermost epidermal layer of seed coat as observed in PJ at S5 (Supplementary Figure S2).

### Identification of Coexpression Modules Regulating Seed Size

In an independent analysis, coexpressed gene sets were identified using WGCNA to investigate the transcriptional networks controlling seed size. A total of 18 modules that grouped genes with similar expression patterns were identified after merging closely related modules (Supplementary Figure S6C; Supplementary Table S10). The module eigengene of each coexpression module was associated with stages of seed development, genotype, and an increase in seed size via calculation of Pearson’s correlation coefficient (Supplementary Figure S6D). Several modules were correlated with more than one developmental stage; however, some modules were specific to just one stage, indicating stage-specific coexpression networks. For example, cyan, royalblue, and brown modules showed high positive correlations with stages S1, S2, and S3, respectively. The list of genes and GO terms associated with each WGCNA module is given in Supplementary Tables S10, S11, respectively. We identified the hub genes in our module of interest using gene connectivity (K_within_) measure. The genes with the top 10% K_within_ in a module were identified as the hub genes in that module.

**FIGURE 4:**
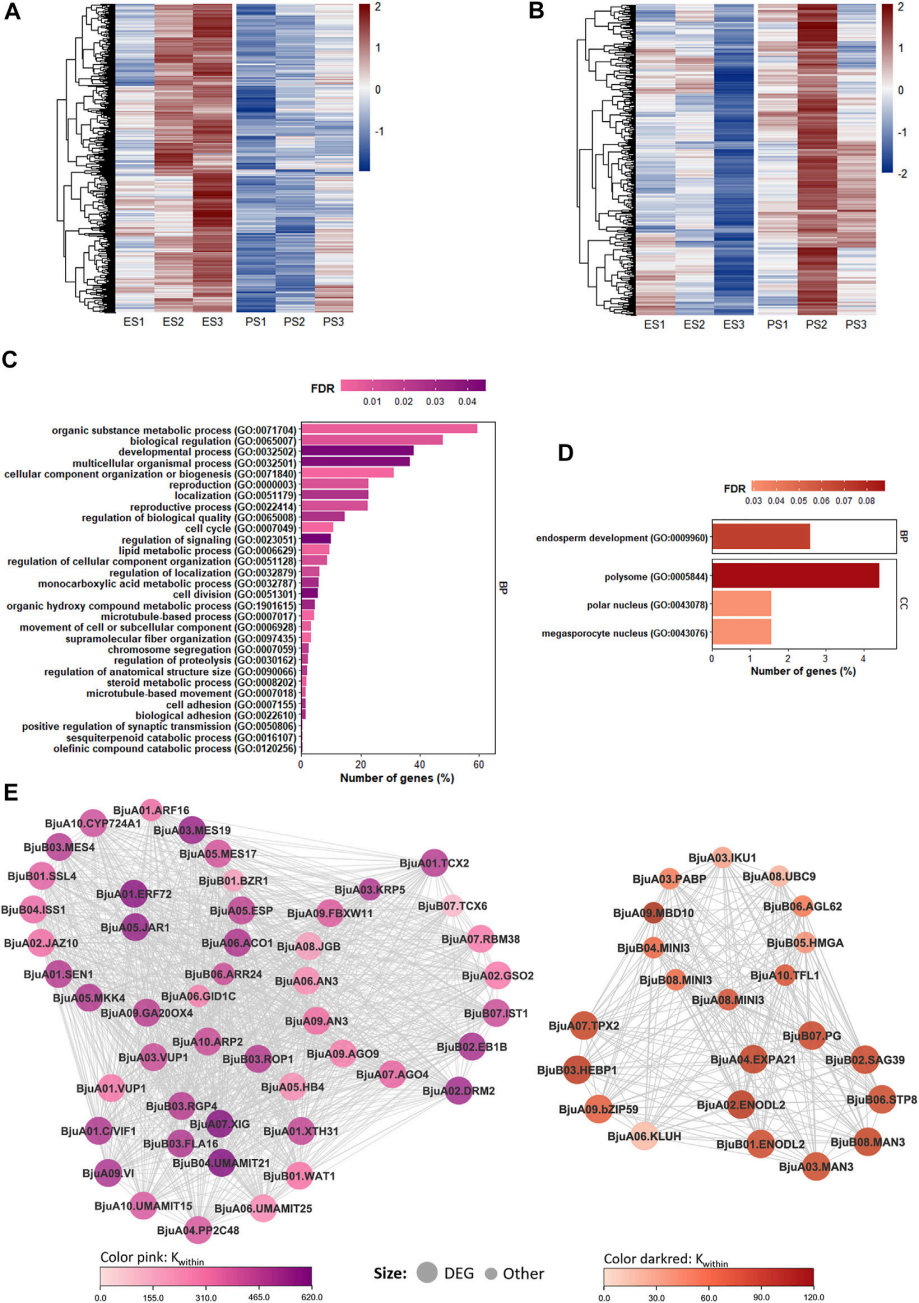
Coexpression modules with the highest correlation with increase in seed size. **(A)** Heatmap showing expression profiles of the pink module. The color scale on the right represents Z-scores. **(B)** Heatmap showing expression profiles of the darkred module. The color scale on the right represents Z-scores. **(C)** GO enrichment (top 30 biological process enriched terms) of the pink module. **(D)** GO enrichment of the darkred module. **(E)** Sub-networks connecting genes related to cell cycle and cell division in the pink module and endosperm development in the darkred module to hub genes in EPJ Tsw QTL. E, EH-2; P, PJ; S1–S3, seed development stages; GO, Gene Ontology; Tsw, thousand seed weight.

Module eigengenes of pink (r^2^ = −0.8, *p*-value = 1e−04) and darkred (r^2^ = 0.79, *p*-value = 1e−04) modules were found to be highly associated with an increase in seed size, and these modules harbored genes with high gene significance for an increase in seed size (Supplementary Figure S6E; Supplementary Table S10). The pink module harbored 2,383 genes with higher expression in EH-2 at the S2 and S3 stages and showed a negative correlation with the increase in seed size (Figure 4A). The K_within_ values in the pink module ranged from 3.9 to 616.6. A total of 250 genes in the pink module including 42 TFs were significantly (padj < 0.05, lfcthreshold = 1) upregulated in EH-2. GO enrichment analysis of pink module revealed a significant overrepresentation of GO terms “reproductive process (GO:0022414),” “cell cycle (GO:0022414),” “cell division (GO:0051301),” and “regulation of anatomic structure size (GO:0090066)” (Figure 4C). Several DEGs identified as regulators of cell proliferation and expansion (Figure 3) also showed up as hub genes in the pink module (Figure 4E). Bju.TCX2 (BjuVaA01g002241, K_within_ = 485.4) was identified as a hub gene in the pink module and was significantly upregulated in EH-2 (Figure 4E). TCX2 restricts cell proliferation via the regulation of genes in the DREAM complex (Clark et al., 2019). Genes involved in small RNA mediated DNA methylation in CHH context; Bju.AGO4 (BjuVaA07g001798), Bju.AGO9 (BjuVaA09g003432), and Bju.DRM2 (BjuVaA02g000576) were upregulated in EH-2, indicating their relevance in the negative regulation of seed size in *B. juncea*. We identified several DEGs involved in hormone signaling in the pink module with Bju.ERF72 (BjuVaA01g004064, K_within_ = 585.1) and Bju.JAR1 (BjuVaA05g000146, K_within_ = 555.02) as the hub genes. Together, the coregulated gene sets in the pink module provide insights on transcriptional programs, restricting seed growth in *B. juncea*.

The darkred module harbored 386 genes with higher expression in PJ at the S2 and S3 stages and showed the highest positive correlation with an increase in the seed size (Figure 4B). The K_within_ values in the darkred module ranged from 4.6 to 114.3. A total of 27 genes in the darkred module including five TFs were significantly (padj < 0.05, lfcthreshold = 1) upregulated in PJ. The GO enrichment of the darkred module revealed an overrepresentation of biological process “endosperm development (GO:0009960)” (Figure 4D). Based on K_within_ scores, we identified DEGs, Bju.ENODL2 (BjuVaA02g001491, K_within_ = 104.2), Bju.HEBP1 (BjuVaB03g001743, K_within_ = 103.59), Bju.EXP21 (BjuVaA04g001209, K_within_ = 99.07), and Bju.TPX2 (BjuVaA07g003244, K_within_ = 95.89) as the hub genes in the darkred module (Figure 4E). Bju.KLUH (BjuVaB03g001743) was significantly upregulated in PJ at S3. KLUH has been reported to increase seed size and oil content in *A. thaliana* seeds (Adamski et al., 2009). The coexpression of these DEGs with key regulators of endosperm development and seed size including Bju.AGL62, Bju.MINI3, Bju.IKU1, and Bju.UBC9 warrants a further investigation of their roles in seed development with respect to regulation of seed size in *B. juncea*.

### Candidate Genes Underlying the Quantitative Trait Locus Governing Seed Size

**FIGURE 5:**
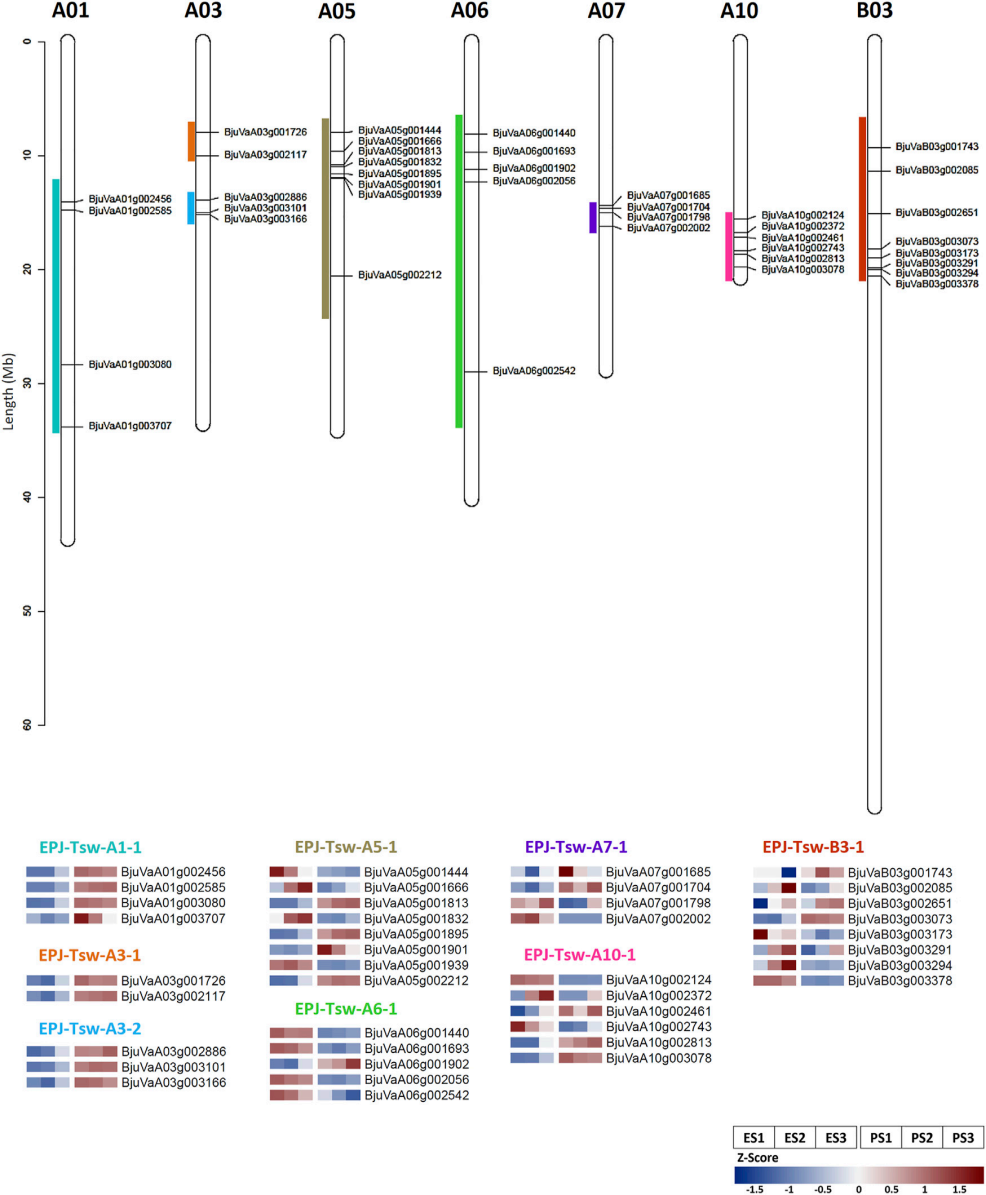
Candidate genes for regulation of seed size in EH-2 and PJ colocalizing the EPJ Tsw QTL. Genomic locations (in EPJ Tsw QTL) and expression profiles (Z-score) of selected candidate genes at stages S1–S3 in EH-2 and PJ. E, EH-2; P, PJ; S1–S3, seed development stages; Tsw, thousand seed weight.

We identified the DEGs colocalizing EPJ Tsw QTL (Dhaka et al., 2017) to further shortlist candidate genes involved in the regulation of the seed size in EH-2 and PJ. A total of 588 genes encompassing eight Tsw QTL exhibited differential expression in the EH-2 and PJ seeds (Supplementary Table S13). We identified a total of 40 DEGs colocalizing the eight EPJ Tsw QTL as candidates for the regulation of seed size on the basis of their association with seed size/weight, cell proliferation and cell wall biogenesis/modification, and/or hub gene status in pink or darkred modules with gene significance for an increase in seed size >±0.7 (Figure 5; Supplementary Table S14). These 40 candidate genes include 15 TFs belonging to the ARF, GeBP, WRKY, EIL, E2F/DP, GRF, CPP, C3H, bHLH, MYB-related, NAC, and DoF TF families. The candidate genes for seed size include genes regulating cell proliferation/division, cell wall biogenesis/modification, solute/hormone transporters, DNA methylation, auxin, cytokinin, ethylene, and JA responsive genes (Supplementary Table S14) highlighting the importance of these pathways in differential regulation of seed size in *B. juncea*.

Several DEGs related to the regulation of cell wall biosynthesis/modification including Dof zinc finger protein DOF3.1 (BjuVaA01g003707), galactosyl transferase family protein XXT3 (BjuVaA10g002813), a CPP family TF endoglucanase 25 (BjuVaA05g001901), EXT21 (BjuVaB03g002651), FLA16 (BjuVaB03g002085), and RGP4 (BjuVaB03g003291) colocalized EPJ Tsw QTL and are candidates for the regulation of seed size. We observed an upregulation of a C3H TF, Bju.CYAB1;4 (BjuVaA01g003080) colocalizing EPJ-Tsw-A1-1 in PJ at S1 and S2, suggesting higher rates of mitotic activity in PJ at stages S1 and S2. An increased expression of CYAB1;4 has been shown to increase seed size in *A. thaliana* (Ren et al., 2019). Several auxin-responsive DEGs including Auxin transporter-like protein 1 (BjuVaA03g002117), Bju.AGO10 (BjuVaA01g002456), Bju.Dof3.1 (BjuVaA03g001726), Bju.ARF2 (BjuVaA06g002542), Bju.IAA22 (BjuVaA06g001440), and Bju.PATL5 (BjuVaB03g003378) colocalized EPJ Tsw QTL and suggest a role of auxin signaling in differential regulation of seed size in *B. juncea*. EPJ-Tsw-A3-2 QTL harbored a cytokinin-responsive gene, a two-component response regulator ARR22-like protein (BjuVaA03g003166) with higher transcript abundance in PJ as a putative candidate for regulation of seed size. A component of the ethylene signaling cascade, Bju.EIL1 (BjuVaB03g003073), was significantly upregulated in PJ at S1 and S2 and colocalize with EPJ-Tsw-B3-1 QTL, suggesting a role of ethylene signaling in the regulation of the seed size. *O. sativa* homolog of Bju.EIL1 has been reported to increase grain size via mutant analysis (Yang et al., 2015). EPJ-Tsw-A10-1 QTL harbored Bju.SWEET15 (BjuVaA10g002461), upregulated in PJ, is one of the candidates for the regulation of seed size. SWEET genes play critical roles in sugar transport from seed coat to developing embryo during seed filling, and a *sweet11;12;15* triple mutant has been shown to reduce dry seed weight in *A. thaliana* (Chen et al., 2015). EPJ-Tsw-A5-1 QTL harbored Bju.FAD3 (BjuVaA05g001444) gene, which was significantly upregulated in EH-2. Silencing of gmFAD3 has been reported to increase seed size in soybean (Singh et al., 2011). Based on these results, it is possible to target these genes for the enhancement of seed size in *B. juncea*.

## Discussion

Seed size is a multigenic trait that is predominantly controlled by cues from maternal and zygotic tissues with some environmental influence (Li and Li, 2016). *B. juncea* holds the maximum diversity in seed size/weight among the cultivated Brassicas, with PJ (Indian gene pool) and EH-2 (east European gene pool) showing high phenotypic contrasts for the trait; however, the mechanism of regulation of seed size in *B. juncea* is poorly understood. QTL analysis for Tsw in the EPJ DH population identified large genomic regions harboring several genes. Furthermore, a large number of small-effect Tsw QTLs were identified in the study (Dhaka et al., 2017), indicating a role of multiple genetic factors in differential regulation of seed size in EH-2 and PJ, which limited the scope of Tsw QTL dissection in the EPJ population using the candidate gene approach. Therefore, a comparative transcriptomics approach was undertaken to identify putative candidate genes and to understand the regulatory mechanisms underlying the trait.

We performed a comprehensive transcriptome profiling of the initial stages of seed development (S1–S3) in EH-2 and PJ. The expression data showed high reproducibility and distinct clustering of seed development stages in the PCA plot, reaffirming the results from the anatomical analysis that embryogenesis progresses at a comparable pace in both large- and small-seeded *B. juncea* lines. We identified 5,974 DEGs including 954 TFs belonging to 51 TF families with an overrepresentation of B3, S1Fa-like, WRKY, and NAC TF families in regulating early seed development in *B. juncea*. The role of these TF families has been established in seed development (Le et al., 2010; Agarwal et al., 2011). Members of several TF families, WRKY, ARR-B, GRF, MYB, ARF, B3, AP2, bHLH, bZIP, E2F/DP, MADS, MIKC-MADS, NAC, CPP, and TCP showed differential expression in EH-2 and PJ in our dataset (Figure 2B). These TF families have been implicated in the control of the seed size and/or organ growth (Yang et al., 2008; Martín-Trillo and Cubas, 2010; Mathew et al., 2016; Li et al., 2019). However, the exact role of this TF in the regulation of seed size remains to be established.

Seed size is mostly regulated via differential cell proliferation and cell expansion in different seed compartments, which influence the final seed size (Savadi, 2018; Li et al., 2019). Differential increase in seed size at different stages in EH-2 and PJ (Figure 1C) and increased cell size and cell number in the epidermal layer of seed coat in PJ seeds sampled at S5 as compared to EH-2 (Supplementary Figure S2) suggested that differential regulation of cell proliferation and cell expansion coordinately explain the observed phenotypic variation in seed size in these lines. It has been hypothesized that common genetic components regulate the size of seeds and other organs via affecting cell size and cell number (Linkies et al., 2010). We observed an upregulation of genes related to cell wall biogenesis and/or expansion including expansins, extensins, XTHs, PMEs, and CeSA (Figure 3; Supplementary Table S3) in PJ, suggesting that higher rates of cell wall biosynthesis and/or expansion in PJ might be responsible for its larger seed size via an increase in cell size. We also observed differential regulation of cell cycle and DNA replication machinery in EH-2 and PJ (Figure 3; Supplementary Table S8). There was an upregulation of CYCB1;4 (BjuVaA01g003080) gene in PJ at stages S1 and S2, indicating that increased cell proliferation at these stages might result in increased seed size in PJ. Altogether, higher cell expansion accompanied by an increased mitotic activity in PJ might explain its larger seed size.

We identified coexpressed gene sets/modules correlated with an increase in the seed size. The GO analysis of modules correlated with an increase in seed size, which suggested an influence of genes involved in the cell cycle, cell division, and endosperm development in the determination of the final seed size in EH-2 and PJ. We identified Bju.TCX2 (BjuVaA01g002241) as hub gene in pink coexpression module, negatively associated with seed size. TCX2 is a homolog of the DREAM complex gene LIN54 and restricts stem cell proliferation in *A. thaliana* root (Clark et al., 2019). The role of the DREAM complex in the regulation of seed size has been hypothesized via transcriptome and coexpression analysis of seed development in *B. rapa* (Niu et al., 2020). Bju.TPX2 was upregulated in PJ and was identified as a hub gene in the darkred module, which is positively correlated with an increase in the seed size. TPX2 is a positive regulator of cell proliferation (Tomaštíková et al., 2020), and its coexpression with genes associated with endosperm development warrants detailed analysis of its role in seed development in *B. juncea* with respect to the determination of seed size.

An interplay of multiple plant hormones, including auxins, cytokinins, gibberellins, brassinosteroids, ethylene, and JA, have been shown to regulate different aspects of seed development including seed size (Savadi, 2018; Li et al., 2019; Hu et al., 2021). Pathway analysis suggested differential hormone regulation in EH-2 and PJ during the S1–S3 stages (Supplementary Figure S5). Additionally, *B. juncea* homologs of several hormonal regulators of seed size in *A. thaliana* and *O. sativa* were differentially expressed in EH-2 and PJ (Supplementary Table S9). We observed that 7 out of 9 ARF family TFs were upregulated in EH-2 (Figure 2B). An auxin-induced JA responsive gene Bju.JAR1 was significantly upregulated in EH-2 and was identified as a hub gene in the pink module (Figure 4E). There is limited evidence of the role of JA signaling in the regulation of seed size. However, a recent study by Hu et al. (2021) has shown that increased levels of JA repress seed size via negative regulation of the integument cell proliferation. We identified genes in the ethylene signaling pathway, Bju.ACO1 and Bju.ERF2, as hub genes in the pink coexpression module (Figure 4E). Ethylene has been shown to influence the seed size in the early stages of seed development in *B. napus* in a dose-dependent manner (Walton et al., 2012); however, the mechanism of control of seed size via ethylene signaling is not well characterized. An overexpression of ERF72 has been reported to inhibit hypocotyl elongation in *A. thaliana* by inhibition of cell elongation and links auxin, brassinosteroid, and ethylene signaling (Liu et al., 2018). Further functional studies on the hub genes in the coexpression networks might reveal novel genes for improving seed size and could be crucial to understanding the regulatory mechanisms underlying the trait.

An integration of the DEG and coexpression datasets with eight EPJ Tsw QTL identified forty candidate genes for engineering seed size in *B. juncea*, including genes regulating cell cycle, cell wall biogenesis/modification, solute/sugar transport, and hormone signaling (Supplementary Table S14). A total of five EPJ Tsw QTL, including EPJ-Tsw-A3-1, EPJ-Tsw-A3-2, EPJ-Tsw-A7-1, EPJ-Tsw-A10-1, and EPJ-Tsw-B3-1, were part of the consensus Tsw QTL detected in four bi-parental DH mapping populations, TD, VH, DE, and EPJ (Dhaka et al., 2017), providing broad application prospects to the 23 candidate genes harbored in these QTL. Eight DEGs identified as hub genes in coexpression modules highly correlated with an increase in seed size and also colocalized EPJ Tsw QTL including Bju.ESP, Bju.MKK4, Bju.AGO4, Bju.CYP72A1, Bju.FLA16, Bju.RGP4, Bju.HEBP1, and Bju.MES4. Some of these genes have not been reported earlier to directly regulate seed size and could be novel candidates for the improvement of the trait.

Direct orthologues of a few candidate genes colocalizing EPJ Tsw QTL have been reported to regulate seed size in *A. thaliana* and other crops; however, their precise roles in the regulation of seed size in *B. juncea* are yet to be established. Auxin response factor Bju.ARF2 (BjuVaA06g002542) was upregulated in EH-2 at stages S1–S3 and colocalized with EPJ-Tsw-A06-1 QTL. Overexpression of the ARF2 gene has been reported to decrease the seed size in *A. thaliana* by restricting cell proliferation and expansion of the integuments (Schruff et al., 2006). *A. thaliana* homolog of Bju.AGO10 (upregulated in PJ, colocalizing EPJ-Tsw-A1-1) has been shown to downregulate ARF2 expression during embryogenesis (Roodbarkelari et al., 2015), but its direct role in the regulation of seed size has not been elucidated. Homologs of two candidate genes identified in the study, ARF2 and SWEET15, have also been reported as Tsw candidates using analysis of putative selective sweeps in *B. juncea* (Kang et al., 2021). Altogether, these genes are promising candidates for the enhancement of the seed size in *B. juncea*; however, they need to be functionally validated to establish their precise roles in the regulation of seed size.

In conclusion, a comprehensive transcriptome profiling of early seed development in *B. juncea* lines EH-2 and PJ revealed possible mechanisms of seed size regulation. A total of 5,746 genes including 954 TFs were differentially expressed. Our results suggest that differential regulation of cell proliferation and expansion as a result of differential expression of genes regulating cell cycle, cell wall biogenesis/modification, solute/sugar transport, and hormone signaling result in observed phenotypic contrasts in seed size in EH-2 and PJ. The study reports candidate genes for differential regulation of seed size in *B. juncea* lines of east European (EH-2) and Indian (PJ) gene pools. The 40 DEGs colocalizing with the eight EPJ Tsw QTL were identified as the most promising candidates for breeding efforts targeted at an improvement of the seed size of the east European gene pool lines of *B. juncea*. The candidate genes identified in the study will be functionally validated in the future to decipher their precise roles in the determination of final seed size/weight and to leverage their potential for improvement of the seed size in *B. juncea*. Overall, the study provides crucial insights to understand the biology of early seed development in *B. juncea*, which we anticipate will aid the improvement of seed-associated traits in oilseed mustard.

## Data Availability

The obtained RNA sequencing data were deposited to the NCBI BioProject public database and are accessible under the accession number PRJNA771260. The BioSample accession numbers for PJ and EH-2 are SAMN22260750 and SAMN22261840, respectively. The GenBank assembly accession of the reference genome used in the study is GCA_015484525.1. The datasets supporting the conclusions of this article are included within the article/Supplementary Material.

## Footnotes

1
http://www.bioinformatics.babraham.ac.uk/projects/fastqc

2
http://planttfdb.cbi.pku.edu.cn

3
https://www.biobam.com/omicsbox

4
http://revigo.irb.hr
