# Supplementary material for: Comparative Analysis of Seed Transcriptome and Coexpression Analysis Reveal Candidate Genes for Enhancing Seed Size/Weight in Brassica juncea
Source: Front Genet. 2022 Feb 24;13:814486. doi: 10.3389/fgene.2022.814486 (PMC8907137; doi:10.3389/fgene.2022.814486)
Supplement: Supplementary file 1 [file DataSheet2.docx]

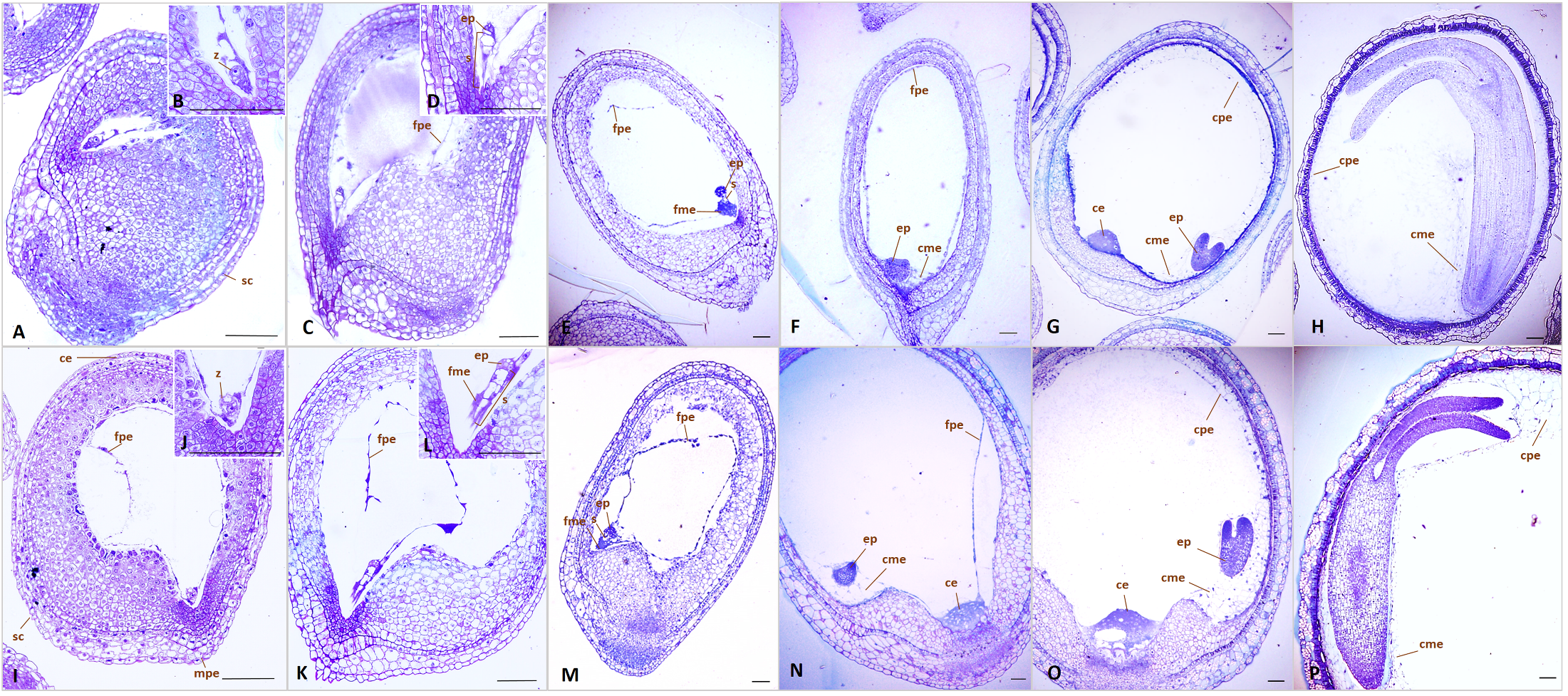


**Supplementary Figure S1** Cross-sections of developing seeds of EH-2 **(A-H)** and PJ **(I-P)** sampled at different stages of seed development (S0-S5); Zygote at S0 (**A, B, I, J**); 2-celled/ 4-celled embryo at S1 (**C, D, K, L**); Globular embryo at S2 (**E, M**); Transition/early-heart stage embryo at S3 (**F, N**) which also marks the stage of endosperm cellularisation; Late-heart stage embryo at S4 (**G, O**) and Bent-cotyledon stage embryo at S5 (**H, P**); Scale bars represent 100 μm; Abbreviations: z, zygote; ep, embryo proper; s, suspensor; mpe, micropylar end; ce, chalazal end; sc, seed coat; fpe, free-nuclear peripheral endosperm; cpe, cellularised peripheral endosperm; fme, free-nuclear micropylar endosperm; cme, cellularised micropylar endosperm; ce, chalazal endosperm


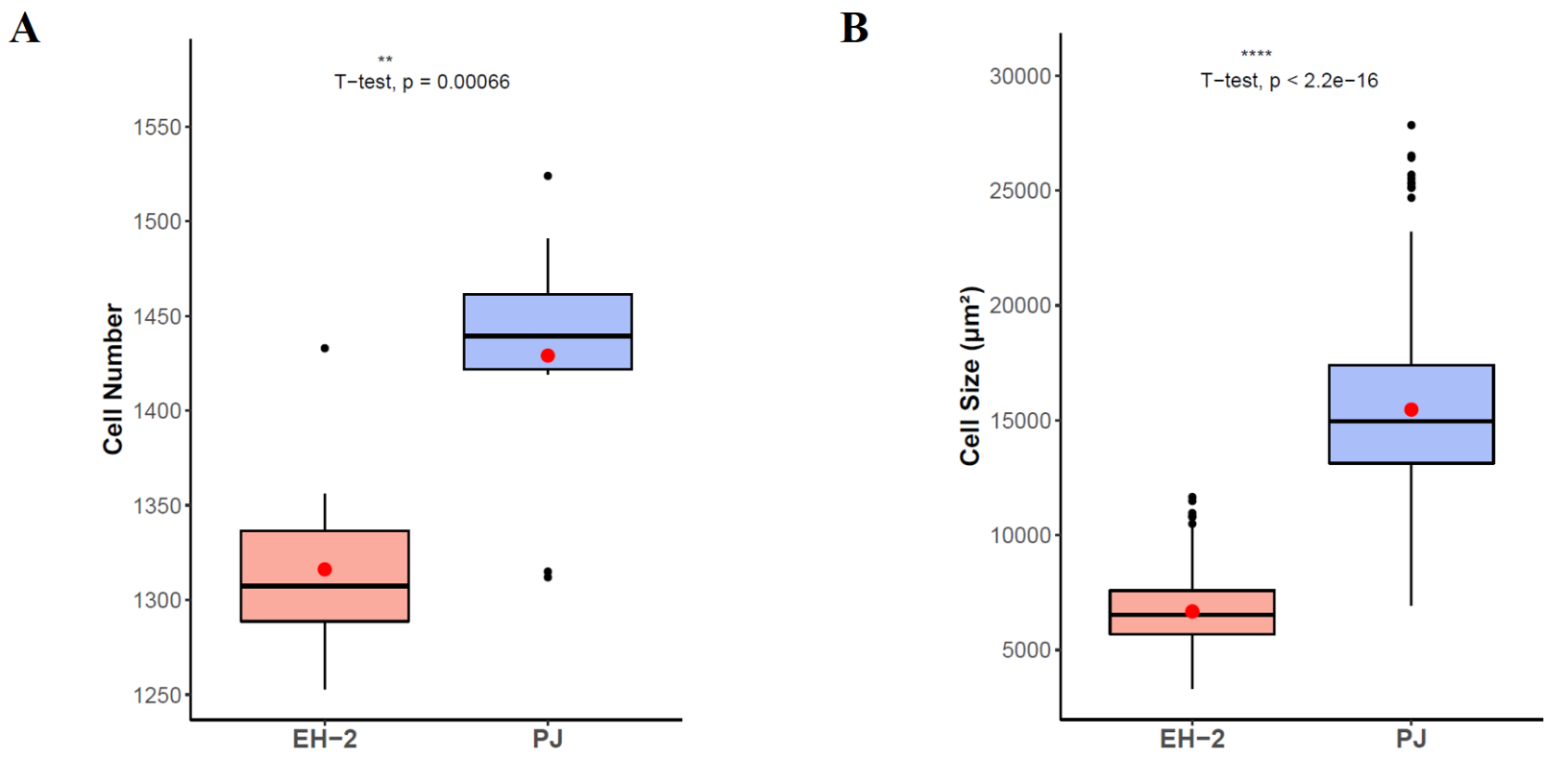


**Supplementary Figure S2** Differences in (**A)** cell size and **(B)** cell number in the seed coat (outermost epidermal layer) of EH-2 and PJ at S5 stage.


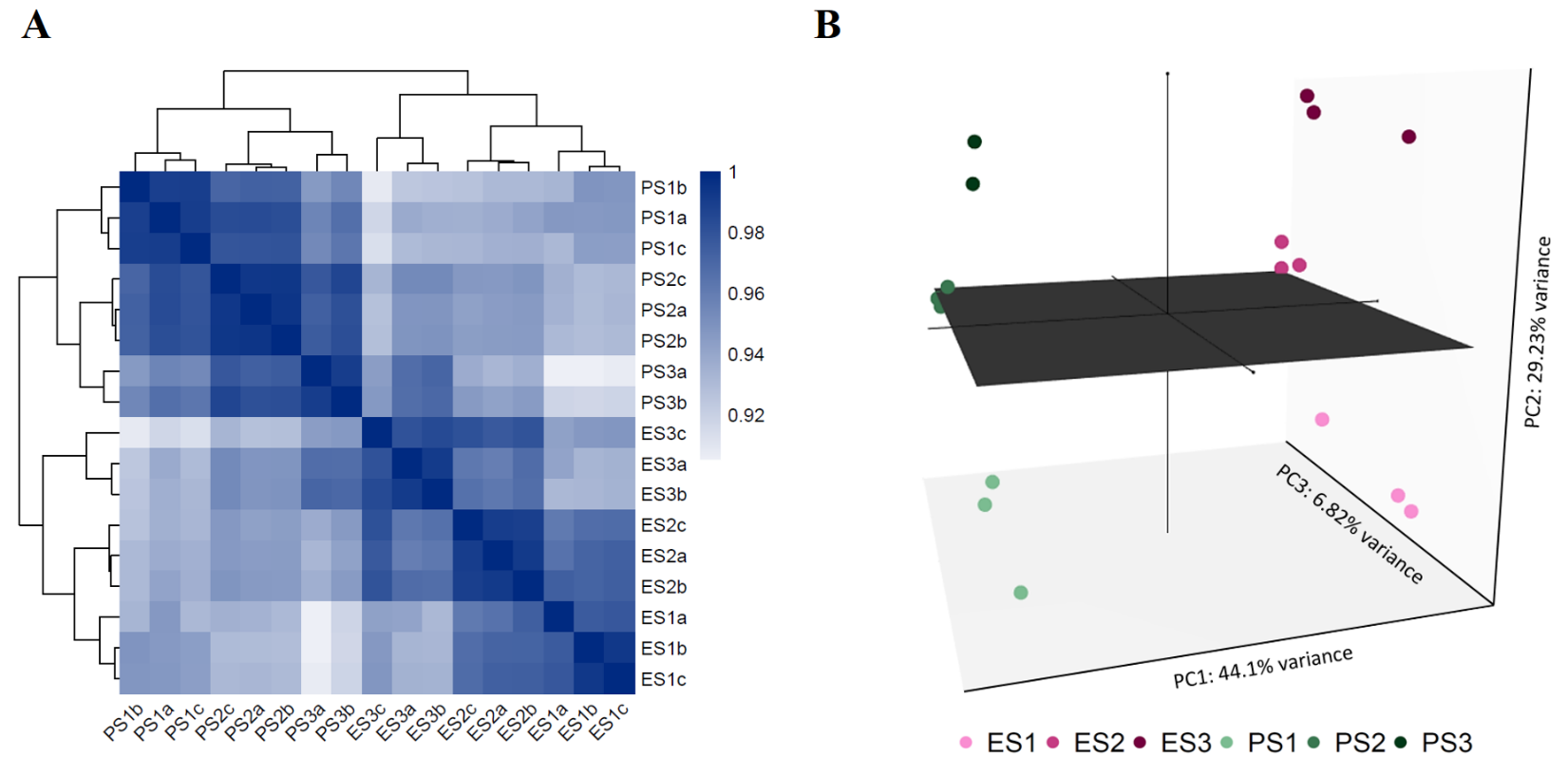


**Supplementary Figure S3** Correlation between the transcriptomes of different stages of seed development in the two *B. juncea* lines; **(A)** Heatmap depicting Pearson correlation coefficients of biological replicates of EH-2 and PJ at stages S1-S3; **(B)** Principal component analysis (PCA) plot showing clustering of transcriptomes of different stages of seed development in EH-2 and PJ. E, EH-2; P, PJ; S1-S3, seed development stages; a-c, 3 biological replicates


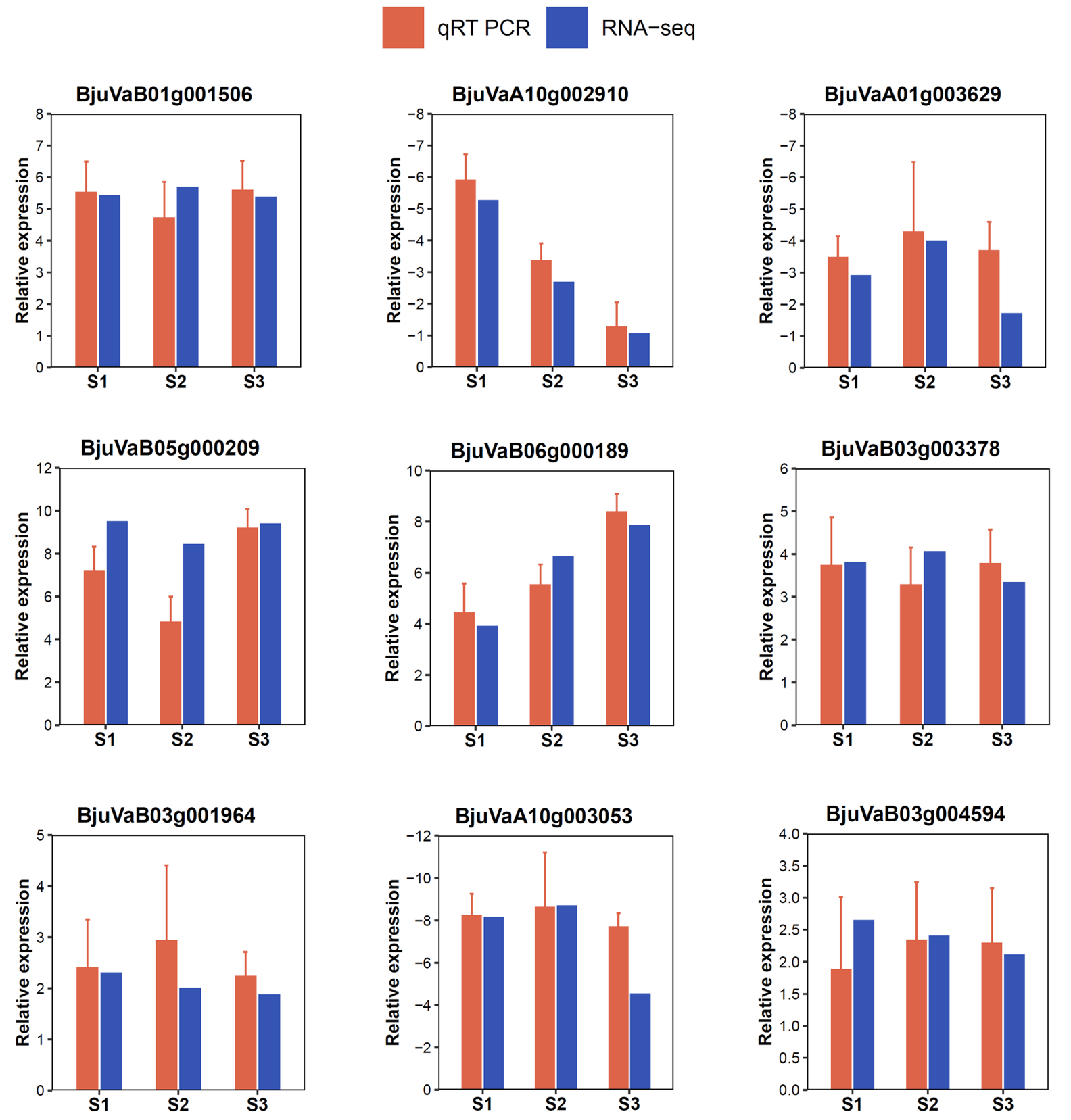


**Supplementary Figure S4** Quantitative PCR validation of selected DEGs. Blue bars depict log2fc obtained from the RNA-Seq data. Error bars represent standard deviation.


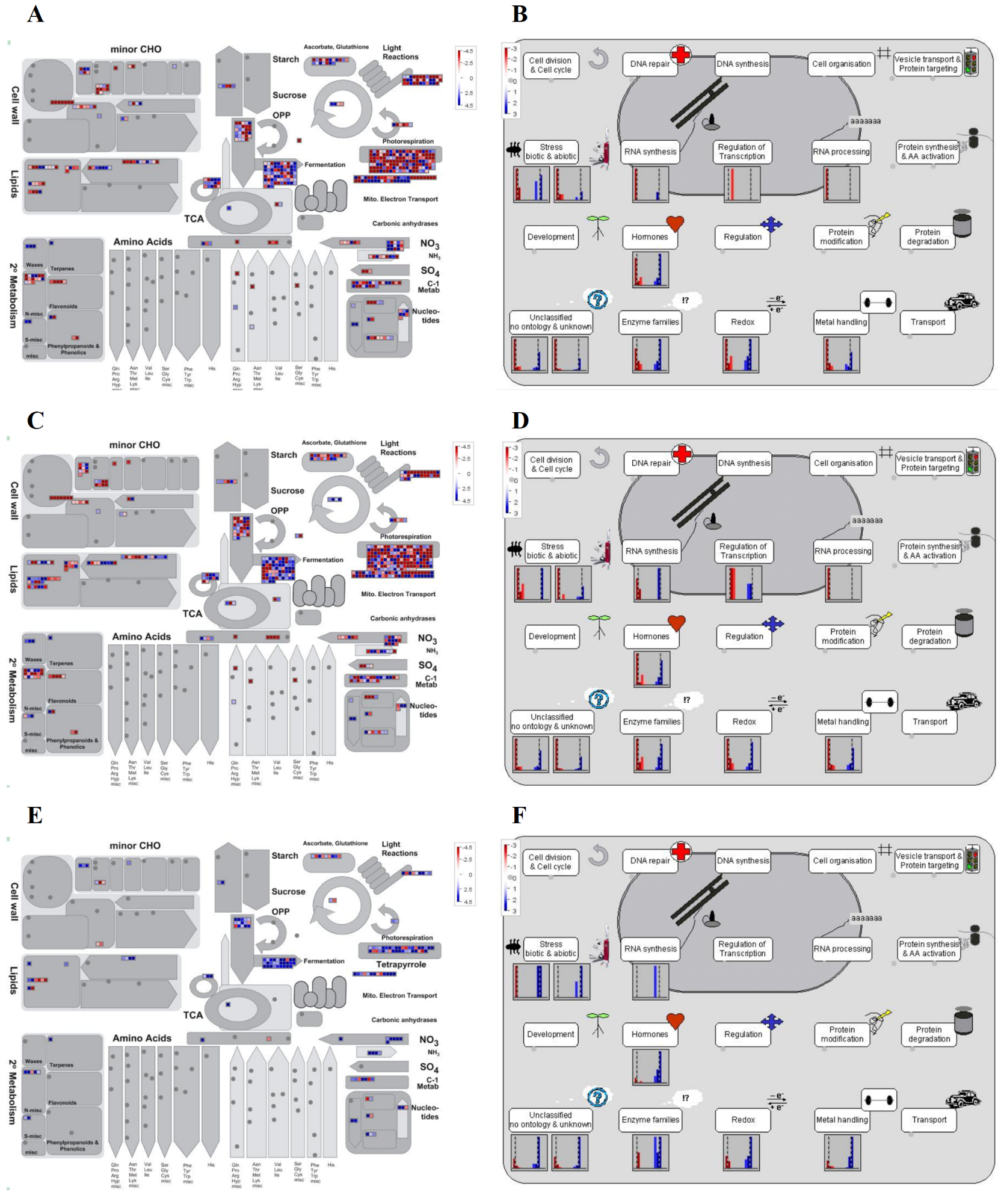


**Supplementary Figure S5** Overview of metabolic pathways **(A, C, E)** and cellular functions **(B, C, F)** differentially regulated in EH-2 and PJ at S1 **(A, B)**, S2 **(C, D)**, and S3 **(E, F)** stages of seed development generated using MapMan. The color scales represent log2 expression values, red and blue colors represent lower and higher expression in EH-2 as compared with PJ, respectively.


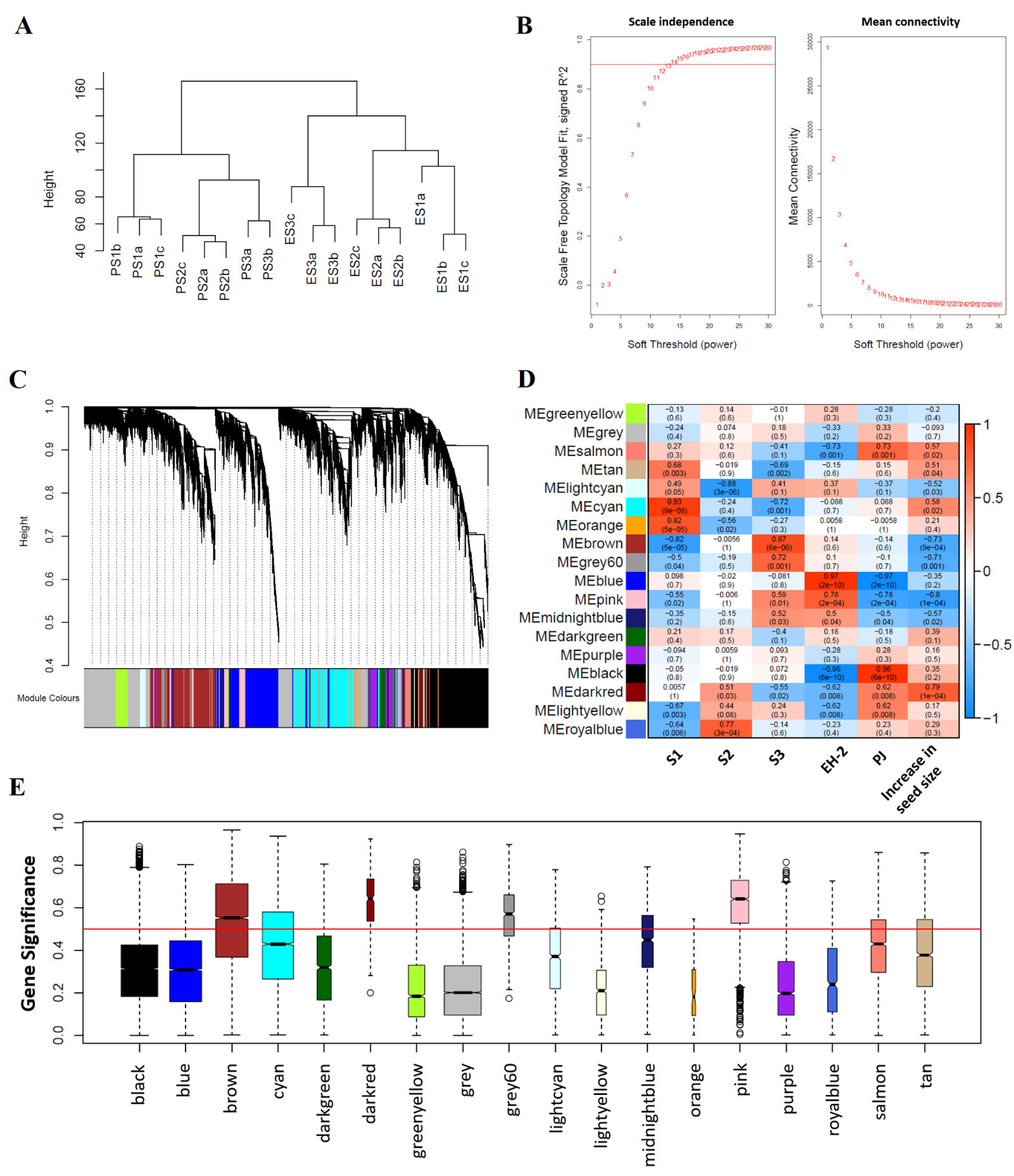


**Supplementary Figure S6** Coexpression network analysis during seed development in EH-2 and PJ; **(A)** Sample clustering to detect outliers; **(B)** Scale independence, Mean connectivity (bicor, maxPOutliers = 0.1); **(C)** Hierarchical clustering tree (dendrogram) of genes based on coexpression network analysis EH-2 and PJ. Each ‘leaf’ (short vertical line) represents one gene. The genes were clustered on the basis of dissimilarity measure (1-TOM). The branches correspond to modules of highly interconnected genes; **(D)** Module relationship with stages (S1-S3), genotypes (PJ and EH-2), seed size and increase in seed size; **(E)** Barplot depicting gene significance (GS) of each module for increase in seed size.
